# Delayed Treatment With Hypothermia Protects Against the No‐Reflow Phenomenon Despite Failure to Reduce Infarct Size

**DOI:** 10.1161/JAHA.112.004234

**Published:** 2013-02-22

**Authors:** Sharon L. Hale, Michael J. Herring, Robert A. Kloner

**Affiliations:** 1 The Heart Institute of Good Samaritan Hospital, University of Southern California, Los Angeles, CA (S.L.H., M.J.H., R.A.K.); 2 Division of Cardiovascular Medicine, Keck School of Medicine, University of Southern California, Los Angeles, CA (R.A.K.)

**Keywords:** ischemia, myocardial infarction, reperfusion

## Abstract

**Background:**

Many studies have shown that when hypothermia is started after coronary artery reperfusion (CAR), it is ineffective at reducing necrosis. However, some suggest that hypothermia may preferentially reduce no‐reflow. Our aim was to test the effects of hypothermia on no‐reflow when initiated close to reperfusion and 30 minutes after reperfusion, times not associated with a protective effect on myocardial infarct size.

**Methods and Results:**

Rabbits received 30 minutes coronary artery occlusion/3 hours CAR. In protocol 1, hearts were treated for 1 hour with topical hypothermia (myocardial temperature ≈32°C) initiated at 5 minutes before or 5 minutes after CAR, and the results were compared with a normothermic group. In protocol 2, hypothermia was delayed until 30 minutes after CAR and control hearts remained normothermic. In protocol 1, risk zones were similar and infarct size was not significantly reduced by hypothermia initiated close to CAR. However, the no‐reflow defect was significantly reduced by 43% (5 minutes before CAR) and 38% (5 minutes after CAR) in hypothermic compared with normothermic hearts (*P*=0.004, ANOVA,* P*=ns between the 2 treated groups). In protocol 2, risk zones and infarct sizes were similar, but delayed hypothermia significantly reduced no‐reflow in hypothermic hearts by 30% (55±6% of the necrotic region in hypothermia group versus 79±6% with normothermia, *P*=0.008).

**Conclusion:**

These studies suggest that treatment with hypothermia reduces no‐reflow even when initiated too late to reduce infarct size and that the microvasculature is especially receptive to the protective properties of hypothermia and confirm that microvascular damage is in large part a form of true reperfusion injury.

## Introduction

Mild hypothermia, in the setting of myocardial infarction, has been shown to be cardioprotective, reducing ischemic injury, in both experimental models^1–4^ and, to a limited extent, the few small clinical trials in which it has been tested to date.^5–7^ However, most experimental studies, including those from our laboratory, show that temperature reduction must occur before coronary artery reperfusion to reduce necrosis.^8–10^ It is also known that hypothermia improves the no‐reflow phenomenon. For example, we have shown that regional cooling of the heart, initiated at 20 minutes into a 30‐minute coronary artery occlusion (CAO), not only reduces infarct size but, more importantly, also reduces the no‐reflow defect to a greater extent than would be expected by the reduction in necrosis alone.^3^

Other evidence that hypothermia protects vasculature beyond infarct size reduction was provided by Götberg and coworkers,^8^ who showed that instituting hypothermia just at reperfusion reduced microvascular obstruction in pig hearts as measured with ex vivo magnetic resonance imaging, although infarct size was not reduced. These studies suggest that the microvasculature may be particularly receptive to protection by hypothermia. This concept has important clinical implications, because no‐reflow occurs in as many as one third of patients who receive percutaneous coronary intervention for ST‐elevation myocardial infarction and no‐reflow is an independent predictor of adverse events, including mortality.^11–13^ For recent reviews, see Brosh et al^12^ and Morishima et al.^13^

It is not known whether initiating hypothermia after reperfusion has any beneficial effect on no‐reflow. In the present study, our aim was to test the effects of hypothermia, specifically focusing on no‐reflow, first when hypothermia was initiated close to reperfusion and then also well after reperfusion, at a time when hypothermia has ceased to be effective in reducing infarct size. In protocol 1, we tested the onset of hypothermia starting at 5 minutes before and 5 minutes after coronary artery reperfusion. When we observed a positive benefit at these time points, we performed protocol 2, in which we tested hypothermia onset at 30 minutes after reperfusion began.

## Methods

The rabbits used in this study were maintained in accordance with the policies and guidelines of the position of the American Heart Association on research animal use (American Heart Association, 1985, http://circres.ahajournals.org/content/57/2/330.long) and the 2011 *Guide to Care and Use of Laboratory Animals* (http://www.aaalac.org/resources/theguide.cfm). The Association for Assessment and Accreditation of Laboratory Animal Care International accredits Good Samaritan Hospital. The protocol was approved by the Institutional Animal Care and Use Committee of Good Samaritan Hospital.

### Experimental Protocols

The methods used for the rabbit model of acute myocardial infarction in our laboratory have been previously described.^14^ Briefly, anesthetized (ketamine plus xylazine), open‐chest male New Zealand White rabbits (2.4 to 3.1 kg) were subjected to 30 minutes of CAO followed by 3 hours of reperfusion. The study consisted of 2 parts, protocol 1 and protocol 2, which were conducted sequentially. The individual treatment was determined after CAO by drawing slips of paper with the treatment group written on them. Protocol 1 consisted of 3 groups. Hearts were randomly treated with topical myocardial hypothermia (ice+water bag placed directly on the heart to achieve a mid‐wall temperature of ≈32°C) 5 minutes before reperfusion or 5 minutes after reperfusion or control, in which hearts remained normothermic (≈38°C). Hypothermia was maintained in the treated groups until 60 minutes of reperfusion. In protocol 2, hypothermia was delayed until 30 minutes of reperfusion and was maintained in the treated group until 90 minutes of reperfusion. Thus hypothermia was maintained in treated rabbits in both protocols for 55 to 65 minutes only, and then spontaneous warming was allowed. A needle‐style thermocouple was placed in the ischemic risk region of each heart to monitor temperature.

At the end of the reperfusion period to assess the extent of no‐reflow, a 4% solution of thioflavin S was injected into the heart of each rabbit through a catheter placed in the left atrial appendage. Thioflavin S is a fluorescent yellow dye that stains intact endothelium of the capillaries and serves as a marker of perfusion.^8,15^ Under ultraviolet light, no‐reflow zones appear as nonfluorescent dark areas, while regions that are perfused appear brightly fluorescent. The coronary artery was then reoccluded and the risk zone was delineated by injecting Unisperse (monastral) blue pigment^16^ via the atrial catheter. Normally perfused myocardium with intact capillaries traps blue pigment while the ischemic risk zone remains pink. The heart was removed from the body, the right ventricle was cut away, and the left ventricle was sliced into 7 or 8 pieces cut perpendicular to the atrioventricular groove. To delineate necrosis, the heart slices were incubated for 15 minutes in 1% buffered triphenyltetrazolium chloride at 37°C. Measurements of the risk zone, no‐reflow zone, and infarct size were calculated as previously described.^14^

### Statistical Analysis

Data were calculated and tabulated using Excel worksheets (Microsoft, Redmond, WA). All data summary and statistical analyses were performed using SAS Version 9.3 (SAS Institute, Cary, NC). Left ventricular weight, infarct size, area at risk, and area of no‐reflow were compared using ANOVA in protocol 1 and *t* test in protocol 2. If ANOVA yielded an F value of <0.05, post hoc differences among means were determined by Tukey's test. Changes in hemodynamic variables over time were analyzed by repeated‐measures ANOVA. ANCOVA was used to test for a group effect on the regression model of no‐reflow with necrotic zone. Data are expressed as mean±SEM.

## Results

### Experimental Animals

A total of 66 rabbits were used. Two animals died of hypotension during reperfusion (protocol 1, hypothermia group). In protocol 1, data are reported on 14 rabbits with hypothermia started 5 minutes before coronary artery reperfusion (H25occ group), 12 rabbits with hypothermia started 5 minutes after reperfusion (H5rep group) and 14 normothermic rabbits (N group). In protocol 2, data are reported on 12 rabbits with hypothermia started at 30 minutes (H30rep group) after reperfusion and 12 normothermic rabbits (N group).

### Myocardial Temperature

**Figure 1.**
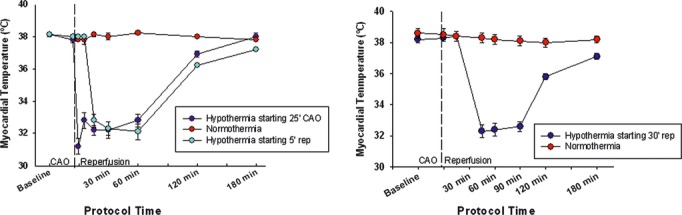
Myocardial risk zone temperatures measured in the ischemic risk area (°C) in protocol 1 (left) and protocol 2 (right). CAO indicates coronary artery occlusion; rep, reperfusion.

Myocardial temperatures are shown in Figure 1. Average baseline temperature was 38.1°C in all groups in protocol 1 and 38.2°C to 38.3°C in protocol 2. Placing the ice/water bag on the heart resulted in a rapid reduction in intramyocardial temperature (1 to 2 minutes) in the risk zone to ≈32°C (target temperature). At the end of the cooling period, the bag was removed and passive rewarming occurred. Hearts in normothermic animals remained within 0.3°C of baseline in both protocols.

## Protocol 1

### Risk Zone and Infarct Size

The ischemic risk zone was similar in the 3 groups and comprised 26±2% of the LV in H25occ hearts, 30±2% in H5rep hearts, and 25±2% in N hearts (*P*=0.22, ANOVA). Although there was a trend for infarct size reduction in H25occ hearts, treatment with hypothermia starting either 5 minutes before or 5 minutes after reperfusion failed to significantly reduce infarct size compared with the N group. Expressed as a percentage of the risk zone, infarct size was 27±3% in H25occ hearts, 42±4% in H5rep hearts, and 34±5% in N hearts (*P*=0.06, ANOVA).

### Reduction of No‐Reflow

**Figure 2.**
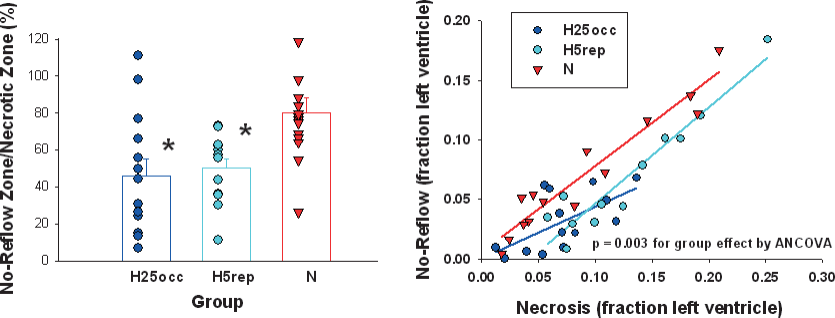
Left, No‐reflow zone expressed as a percentage of the necrotic zone. Both groups had a reduction in the extent of no‐reflow compared with the normothermic group (*P<0.05, ANOVA/Tukey's). Right, Scatterplot of the relationship between necrosis and no‐reflow (both expressed as a fraction of the left ventricle) in the 3 groups. Note that the lines of regression for the 2 hypothermic groups lie below that of the N group, showing that for any given amount of necrosis, there was less no‐reflow in the hypothermia groups.

Although infarct size was not significantly altered by hypothermia, the extent of no‐reflow area within the necrotic zone was significantly reduced, both when hypothermia was initiated 5 minutes before and when hypothermia was initiated 5 minutes after reperfusion (Figure 2, left). Started at 25 minutes of occlusion, hypothermia treatment reduced no‐reflow in the necrotic zone by 43% compared with the normothermic value, and when started at 5 minutes after reperfusion, by 38%. ANCOVA revealed a significant effect of hypothermia on the relationship between the extent of necrosis and the amount of no‐reflow that developed (*P*=0.003, Figure 2, right). On average, for any given extent of necrosis, less no‐reflow developed in the 2 treated groups.

## Protocol 2

### Risk Zone, Infarct Size, and No‐Reflow

**Figure 3.**
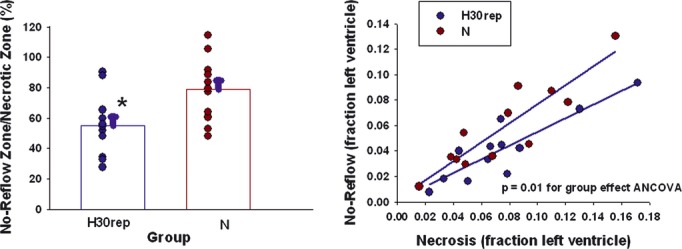
Left, No‐reflow zone expressed as a percentage of the necrotic zone (*P<0.05, *t* test). Right, Scatterplot of the relationship between necrosis and no‐reflow (both expressed as a fraction of the left ventricle) in the 2 groups (*P*=0.01 for group effect, ANCOVA). Note that the line of regression for the hypothermic group lies below that of the N group, showing that for any given amount of necrosis, there was less no‐reflow in the hypothermia group.

In this arm, risk zone comprised 22±2% of the LV in H30 hearts and 25±3% in N hearts (*P*=0.30). Infarct size, expressed as a percentage of the risk zone, was 33±3% in H30 hearts and 30±4% in N hearts (*P*=0.58). Despite the fact that hypothermic treatment failed to reduce myocyte necrosis, no‐reflow in the necrotic zone was reduced by 30% from 79±6% in N hearts to 55±6% in treated hearts (*P*=0.008, Figure 3 left). Analysis of the relationship between the extents of necrosis and no‐reflow revealed a significant reduction by hypothermia (*P*=0.01, Figure 3, right).

### Heart Rate and Blood Pressure in Protocol 1 and Protocol 2

**Figure 4.**
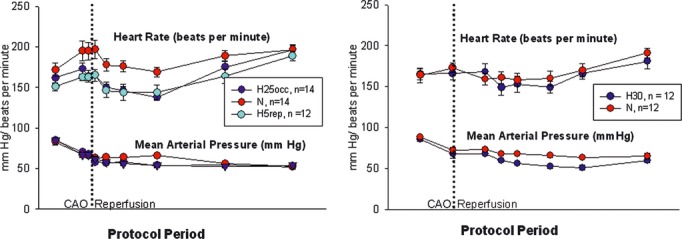
Left, Heart rates and mean arterial pressures (MAPs) in protocol 1. Heart rate, *P*=0.002 for group effect and *P*=0.04 for time effect; MAP *P*=0.001 for time effect, *P*=ns for groups effect. Right, Heart rates and MAPs in protocol 2. Heart rate, *P*=ns for group and time effects; MAP *P*=0.003 for time effect, *P*=ns for group effect. Protocol period=30 minutes of CAO and 180 minutes of reperfusion.

In protocol 1, heart rates were similar at baseline but were reduced in the 2 hypothermic groups starting at the time of treatment (*P*=0.002 for group effect and *P*=0.04 for time effect, repeated‐measures ANOVA). Heart rates in these groups returned to the N values by the end of the reperfusion period. Mean arterial pressures decreased after CAO as is ordinarily seen in this model; however, there were no differences among groups (Figure 4, left). In protocol 2, heart rates were similar in both groups throughout the protocol. Mean arterial pressures decreased after CAO but were not significantly different between groups (Figure 4, right).

## Discussion

**Figure 5.**
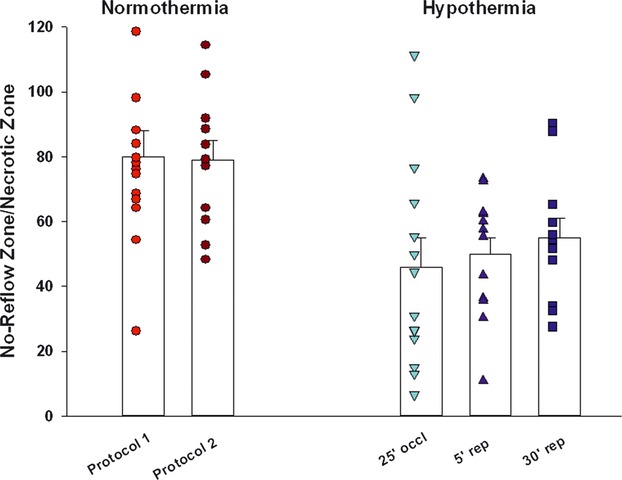
No‐reflow zone, expressed as a fraction of the necrotic zone, for the different times of hypothermia treatment onset in the 2 protocols. rep indicates reperfusion.

Data from this study suggest that hypothermia therapy, instituted near the time of reperfusion or even at 30 minutes after reperfusion, ameliorates microvascular damage, reducing the anatomic zone of no‐reflow. There was a time of treatment effect in that the earlier onset of hypothermia (5 minutes before reperfusion) resulted in the greatest protection, but waiting until 30 minutes after reperfusion to initiate hypothermia still resulted in a significant 30% reduction in the extent of no reflow (Figure 5). This protection occurs in the absence of an effect on infarct size and suggests that the vasculature may be especially receptive to hypothermic protection. This is important because reducing no‐reflow increases blood flow to necrotic areas. Increased microvascular perfusion may improve scar healing and decrease infarct expansion and left ventricular remodeling.^17^

Heart rates were lowered by hypothermia in protocol 1. However, we know from a previous study that benefit conferred by hypothermia is independent of a reduction in heart rate. When hearts were paced to normothermic values, infarct size was still reduced by hypothermia.^18^

Previous studies in rabbits,^3,10,19^ pigs,^1,8^ and sheep^20^ have shown that in addition to reducing myocyte necrosis, hypothermia started before or early into the period of ischemia improves regional perfusion after reperfusion. However, hypothermia started close to the time of reperfusion fails to reduce infarct size in most,^8–9^ but not all,^21^ experimental studies. Our data are consistent with those described by Götberg and coworkers, who tested the effect of a rapid intravenous infusion of cold saline solution into pigs just after reperfusion. Started at this time point, hypothermia did not reduce infarct size, but microvascular obstruction, as assessed by magnetic resonance imaging, was significantly less in hypothermia‐treated pigs.^8^ Data from our present study extend these findings and suggest that the benefit of hypothermia is observed even when onset of hypothermia is delayed until 30 minutes after reperfusion.

The mechanism(s) for the protective effect of hypothermia on myocytes and vasculature has not been elucidated. No‐reflow is characterized by hemorrhage, endothelial swelling, and blebs that appear to obstruct the lumen of microvessels. The present study was not designed to determine the mechanism for the effects of hypothermia on the vasculature. Hypothermia may protect vasculature by reducing the release of cytokines or other inflammatory factors. It has been suggested that one mechanism for the reduction in necrosis when tissue is cooled during the ischemic period is a lessening in reactive oxygen species release at the point of reperfusion. For example, Zar and coworkers tested the effects of mild hypothermia in an isolated rat‐liver model of ischemia–reperfusion and showed that hypothermic perfusion reduced the formation of reactive oxygen species at reperfusion and improved postischemic vascular resistance.^22^ Because the release of free radicals occurs within seconds to minutes after restoration of blood flow, it is apparent from our study and that of Götberg et al^8^ that the benefit that hypothermia confers on the vasculature is independent of an effect on reactive oxygen species generated at the time of reperfusion, because hypothermia was protective even when instituted relatively late after reperfusion.

### Clinical Implications

The importance of reducing no‐reflow in patients has become evident (see Orn et al^23–24^ and Rezkalla et al^23–24^for reviews). The no‐reflow phenomenon occurs in up to a third of all patients who receive percutaneous coronary intervention for ST‐elevation myocardial infarction, and the extent of no‐reflow in patients is an independent predictor of adverse cardiac events including mortality.^11–13^ Opening the occluded epicardial vessel via thrombolysis or percutaneous transluminal coronary angioplasty is the most important therapeutic maneuver to achieve infarct size reduction in patients. However, reducing subsequent microvascular obstruction is now an additional clinical goal. The no‐reflow zone is present primarily in areas that are already necrotic. However, once reperfusion has commenced, there is a progressive expansion of the no‐reflow zone over time.^17^ Reducing microvascular obstruction may improve blood flow to necrotic areas and allow better delivery of pharmacologic agents to the myocardium. In addition, it may improve healing by decreasing infarct expansion and left ventricular remodeling.^24–25^ Although it is not possible to directly translate our results into the possible effect in humans, it is probable that any reduction in no‐reflow in humans would result in better outcome. Rezkalla and coauthors^24^ showed data indicating that patients with severely impaired myocardial blush grade after percutaneous coronary intervention had increased congestive heart failure, cardiogenic shock, and death within 30 days compared with those patients who had normal or near‐normal blush grade.

Attempts have been made to reduce no‐reflow pharmacologically using drugs such as adenosine, nitroprusside, nicardipine, and nicorandil with varying results. Verapamil has been shown to be effective in some small trials. Abciximab also appears to provide some benefit. However, a definitive pharmacologic approach has yet to be established. Mild hypothermia has been tested in a few small previous^5–7,26^ and ongoing^27–28^ trials as an intervention concomitant with percutaneous transluminal coronary angioplasty. The primary end points of these studies are infarct size reduction and fewer adverse clinical events. Although improved no‐reflow will presumably be associated with a reduction in necrosis, it is uncertain whether these studies will show an independent benefit on microvascular obstruction.

### Study Limitations

In this study, hypothermia was achieved by topical application of ice/water on the heart, a technique that is not clinically applicable. In addition, the rabbit ventricular wall is thin (1 to 2 mm), allowing for a very rapid reduction in temperature, something that is not possible in humans. However, other techniques are available for reducing blood and heart temperatures in humans. For example, a recently developed cooling‐suit method currently being tested^27^ can reduce temperature in humans by 3°C in 30 minutes. Cooling has also been achieved using endovascular heat‐exchange catheters, placed into the inferior vena cava or other major vein,^29^ and by surface cooling with temperature‐controlled blankets and ice packs.^30^

### Summary

These studies provide evidence of the benefits of mild hypothermia treatment after ischemia–reperfusion when it is initiated close to the time of reperfusion or even 30 minutes after reperfusion. Infarct size is not reduced by hypothermia when initiated this late. However, microvasculature appears to be preferentially protected. The induction of therapeutic hypothermia may be of benefit in reducing the extent of no‐reflow in the heart, even if started after reperfusion by percutaneous coronary intervention or thrombolysis. Because no‐reflow is an important predictor of outcome after myocardial infarction, hypothermia treatment may improve clinical outcome.
